# Forward Individualized Medicine from Personal Genomes to Interactomes

**DOI:** 10.3389/fphys.2015.00364

**Published:** 2015-12-09

**Authors:** Xiang Zhang, Jan A. Kuivenhoven, Albert K. Groen

**Affiliations:** ^1^Department of Pediatrics, Center for Liver Digestive and Metabolic Diseases, University of Groningen, University Medical Center GroningenGroningen, Netherlands; ^2^Section Molecular Genetics, Department of Pediatrics, University of Groningen, University Medical Center GroningenGroningen, Netherlands; ^3^Department of Laboratory Medicine, Center for Liver Digestive and Metabolic Diseases, University of Groningen, University Medical Center GroningenGroningen, Netherlands

**Keywords:** personalized medicine, interactome, gene regulatory networks (GRN), protein-protein interaction (PPI), genome-scale metabolic models, integrative genomics, network medicine

## Abstract

When considering the variation in the genome, transcriptome, proteome and metabolome, and their interaction with the environment, every individual can be rightfully considered as a unique biological entity. Individualized medicine promises to take this uniqueness into account to optimize disease treatment and thereby improve health benefits for every patient. The success of individualized medicine relies on a precise understanding of the genotype-phenotype relationship. Although omics technologies advance rapidly, there are several challenges that need to be overcome: Next generation sequencing can efficiently decipher genomic sequences, epigenetic changes, and transcriptomic variation in patients, but it does not automatically indicate how or whether the identified variation will cause pathological changes. This is likely due to the inability to account for (1) the consequences of gene-gene and gene-environment interactions, and (2) (post)transcriptional as well as (post)translational processes that eventually determine the concentration of key metabolites. The technologies to accurately measure changes in these latter layers are still under development, and such measurements in humans are also mainly restricted to blood and circulating cells. Despite these challenges, it is already possible to track dynamic changes in the human interactome in healthy and diseased states by using the integration of multi-omics data. In this review, we evaluate the potential value of current major bioinformatics and systems biology-based approaches, including genome wide association studies, epigenetics, gene regulatory and protein-protein interaction networks, and genome-scale metabolic modeling. Moreover, we address the question whether integrative analysis of personal multi-omics data will help understanding of personal genotype-phenotype relationships.

## 1. Introduction

Humans share the same genes but do not have identical DNA sequences. The latest 1000 Genomes Project reported over 84,000,000 single nucleotide polymorphisms (SNPs), 3,000,000 short insertions/deletions, and 60,000 structural variants in 2504 subjects from 26 populations, by applying whole genome sequencing as well as exome sequencing and microarray genotyping technologies (1000 Genomes Project Consortium et al., 2015). While there are large differences in the presence of both rare and common variants, it has been reported that every subject carries around 250–300 loss-of-function variants that lead gene products to having less or no function (1000 Genomes Project Consortium et al., 2010, 2012; UK10K Consortium et al., 2015). Nowadays, whole genome sequencing allows the determination of the entire DNA sequence of an individual, and the resulting genomic information is believed to enable prediction of disease risk and optimization of treatment outcome (Sadee, 2011). In practice, predicting disease phenotypes from genetic sequences is extremely challenging because the genotype-phenotype relationship is far more complex than anticipated. A single gene can be associated with multiple disease phenotypes while a single disease phenotype can be caused by mutations in multiple genes (Barabási et al., 2011). Importantly, mutations do not have identical effects on individuals due to the individual variation in interaction between genes, proteins, metabolites and environmental factors (Barabási et al., 2011; Kathiresan and Srivastava, 2012).

**Figure 1:**
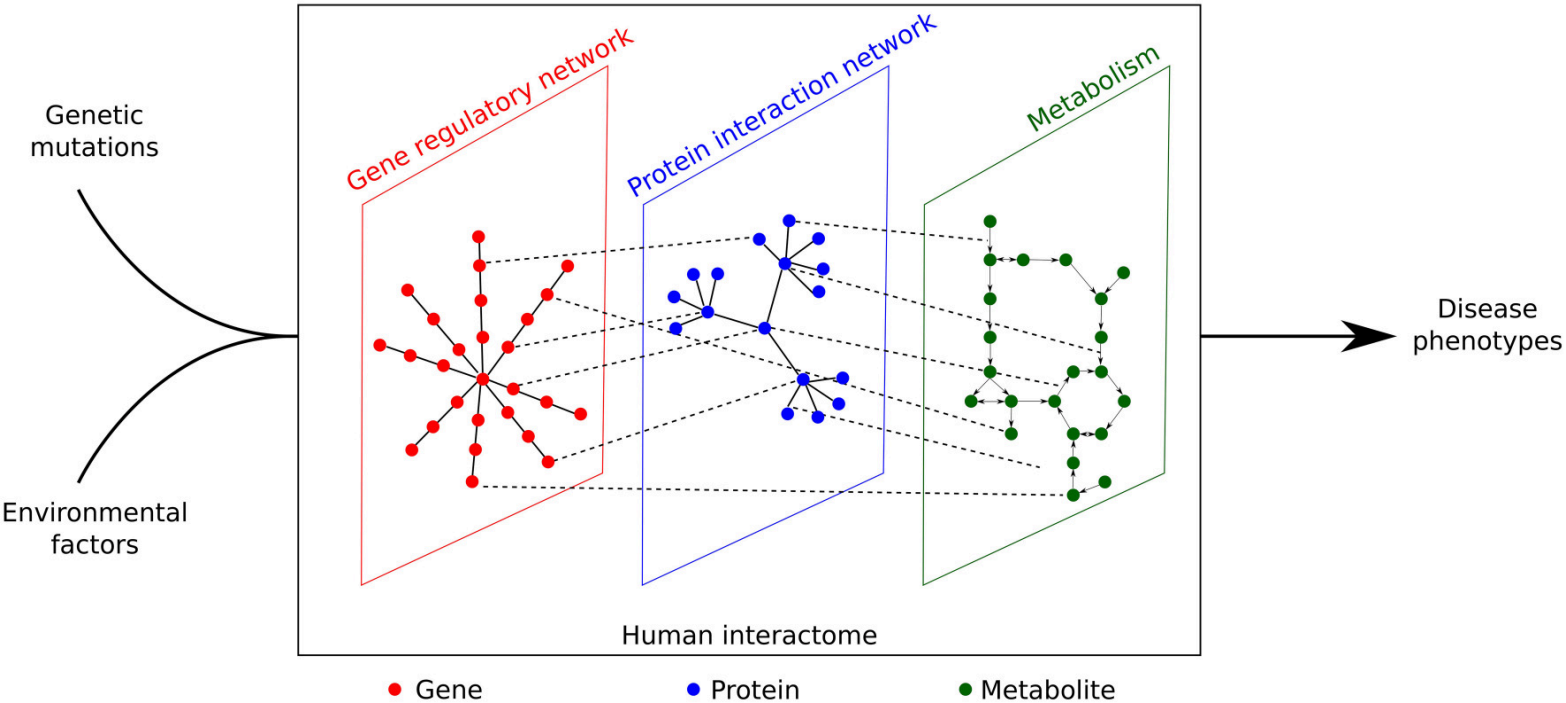
**Genetic mutations and environmental effects can only lead to disease phenotypes through perturbation of the human interactome, which is a complex network constituted by gene regulatory network, protein interaction network, and metabolism**.

The complete set of (physical) interactions between molecules, such as genes, proteins and metabolites is known as the interactome (Cusick et al., 2005). In this review, we focus on the interactome in human cells. If we consider genome sequences as stills and phenotypes as a movie, then there must be a biological system which serves as a projector. It is indeed proposed that the interactome that acts as the projector and eventually translates the phenotypic effects determined by both genotypes and environmental factors (Figure 1). Vidal et al. (2011), Emmert-Streib et al. (2014) proposed that most disease phenotypes may be caused by the perturbation of the interactome, in which the products of disease genes were found to interact with each other and cluster as modules (Ghiassian et al., 2015; Menche et al., 2015). These disease modules may overlap each other, explaining the shared associated genes and clinical symptoms of different diseases (Ghiassian et al., 2015; Menche et al., 2015).

To understand the projector function of the interactome, one must capture all molecular components involved in cellular functions. With the rapid development of omics technologies, it is now possible to readily profile up to 19,797 protein-coding genes, 79,795 protein-coding transcripts, 30,057 proteins, and 4229 metabolites (Psychogios et al., 2011; Harrow et al., 2012; Kim et al., 2014). Since individuality is present in the genomes, epigenomes, transcriptomes, proteomes, and metabolomes, each cell type in every human subject will have a different interactome (Feinberg et al., 2010; Montgomery and Dermitzakis, 2011; Suhre et al., 2011; Forler et al., 2014). In contrast to non-individualized medicine, personalized medicine attempts to address such subject-specific differences with respect to diagnosis and treatment (Topol, 2014). This review aims to give an overview of bioinformatic and network modeling approaches that can be used to develop individualized medicine.

## 2. Genome-wide association studies, epigenetics and individualized medicine

Genome-wide association studies (GWAS) have identified a great number of common single nucleotide polymorphisms (SNPs) that are statistically associated with complex disease phenotypes. The National Human Genome Research Institute (NHGRI) GWAS catalog (www.genome.gov/gwastudies/) includes 1751 curated publications of 11,912 SNPs (Welter et al., 2014). Besides disease-associated SNPs, GWAS also identified SNPs associated with drug efficacy and toxicity, fueling the development of pharmacogenomics and guiding individualized therapies (Sadee, 2011; Crews et al., 2012; Low et al., 2014). The Pharmacogenomics Knowledgebase (PharmGKB, http://www.pharmgkb.org/) (Hewett et al., 2002; Altman, 2007) is a literature-based database which provides useful annotations on genes involved in pharmacokinetics (how the drug is absorbed, distributed, metabolized and eliminated) and pharmacodynamics (how the drug acts on its target and its mechanism of action). In the current release of PharmGKB, curated evidence for 1073 human genes involved in drug response is present.

Epigenetics has been shown to play a key role in the crosstalk between environment and genome, pointing toward the notion that epigenetic marks might explain in part the role of the environment in disease development (Bjornsson et al., 2004; Rivera and Ren, 2013). Major epigenetic alterations include DNA methylation, histone modification, and chromatin remodeling (Rasool et al., 2015). A total number of 127 reference human epigenomes are available on the website of the Roadmap Epigenomics Project (http://www.roadmapepigenomics.org/), including epigenetic landscapes of 111 primary cell and tissue types as well as 16 cell lines (Roadmap Epigenomics Consortium et al., 2015). Due to epigenetic modifications, cells can exhibit different phenotypes in response to various environmental factors, such as nutritional changes and oxidative stress. Feinberg (2007) defined this ability as phenotypic plasticity, whose abnormality is linked to diseases, such as cancers, neurodegenerative and autoimmune disorders (Howell et al., 2009). By integrating GWAS SNPs with epigenetic annotations, Farh et al. (2015) identified that 90% of potentially causal variants of autoimmune diseases are non-coding and 60% map to enhancers of immune cells.

**Figure 2:**
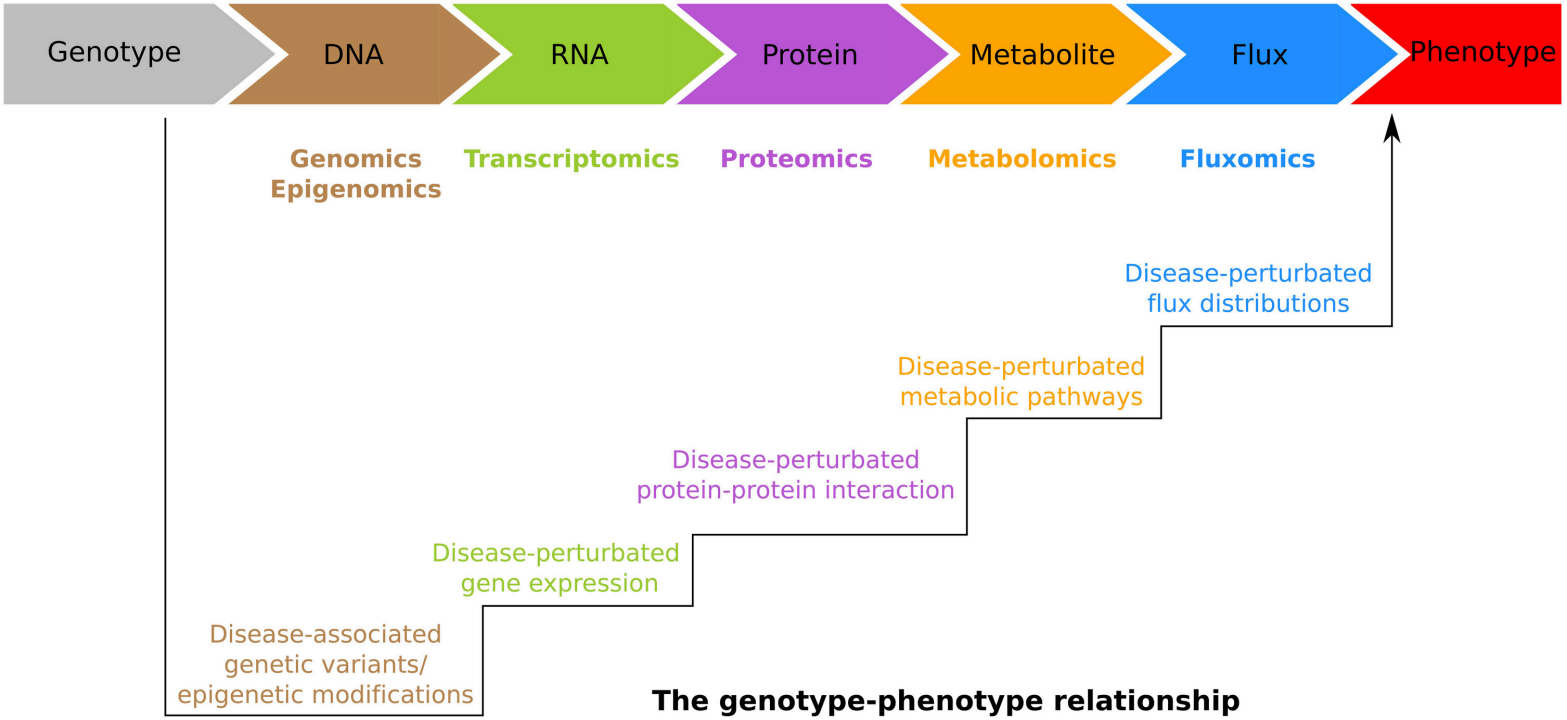
**The genotype-phenotype relationship is hierarchically bridged by DNA, RNA, protein, metabolite and flux**. These molecules are profiled in the genomics, epigenomics, transcriptomics, proteomics, metabolomics, and fluxomics, respectively. Bioinformatics and systems biology approaches try to translate these omics data sets into unified knowledge. In particular, from genomics and epigenomics, one attempts to identify the disease-associated genetic/epigenetic alterations. From transcriptomics, proteomics, metabolomics, and fluxomics, one aims to identify the genes, proteins, pathways, and the flux distributions involved in disease pathogenesis.

In general, information deriving from GWAS (Table 1) and epigenetics provide possible etiological pathways rather than the exact molecular mechanisms underlying diseases. Burke and Korngiebel (2015) pointed out that although dramatic progress has been made in genomics research, there is still a gap between genomic knowledge and clinical application. To fill such gap, an accurate understanding of the genotype-phenotype relationship, which is hierarchically bridged by DNA, RNA, protein, metabolite and flux, must be developed (Figure 2). The integrative personal omics profile (iPOP) study (Chen et al., 2012) was the first example of individualized medicine attempting to overcome the gap by combining omics data sets. Over a 14-month period which also included two viral infections (HRV: human rhinovirus and RSV: respiratory syncytial virus), dr. Michael Snyder not only profiled his whole genome, but also the transcriptomes of his PBMCs (Peripheral Blood Mononuclear Cells) at 20 different time points, proteomes from PBMCs and serum across 14 time points, and metabolomes of his serum sampled 17 time points, respectively. Integration of the data sets revealed the great potential of the individualized approach. In particular, the genetic variant information of dr. Snyder indicated that he is at risk for developing coronary artery disease, basal cell carcinoma, hypertryglyceridemia, and type 2 diabetes, At the same time, he was found carrying variants that are associated with response to glucose lowering drugs, rosigitazone and metformin. Interestingly, his time series measurements of transcriptome, proteome, and metabolome across healthy states, response to RSV infection, and recovery, enabled the authors to identify an alteration of the insulin signaling response following the RSV infection (Chen et al., 2012).

**Table 1:** **Major SNP-trait association databases**.

**Name**	**Link**	**References**
NHGRI GWAS Catalog	www.genome.gov/gwastudies/	Welter et al., 2014
PharmGKB	http://www.pharmgkb.org/	Hewett et al., 2002
GWASdb	http://jjwanglab.org/gwasdb	Li et al., 2012
GWAS Central	http://www.gwascentral.org/	Beck et al., 2014
HuGE Navigator	http://www.hugenavigator.net/HuGENavigator/home.do	Yu et al., 2008
dbGaP	http://www.ncbi.nlm.nih.gov/gap	Tryka et al., 2014
VaDE	http://bmi-tokai.jp/VaDE/	Nagai et al., 2015

The iPOP study also provided us with some important insights on omics-based individualized medicine. First of all, as sequencing technologies vary considerably from each other due to sensitivity, accuracy, coverage and resolution, the measurements may contain systematic errors. Fortunately, since the human genome is constant over time, profiling with multiple DNA sequencing technologies is a way to improve the accuracy of genetic variant detection in an individual genome. As shown in the iPOP study (Chen et al., 2012), a genetic variant in the protein-coding genes can be trusted, if it is captured by the whole genome sequencing as well as whole exome sequencing. Same as above, we can also trust a genetic variant in the non protein-coding genes, if it is identified by different whole genome sequencing platforms. In contrast to the genome which is static, transcriptome, proteome, and metabolome are more dynamic and changes in their patterns represent the most valuable information for individualized medicine. To minimize systematic errors, the personal transcriptomes, proteomes, and metabolomes should be measured with standardized high-throughput methods at different time points and compared longitudinally. The longitudinal design also allows to perform statistical analysis with a single sample through applying well-established time-series data analysis techniques, such as Fourier spectral analysis and autocorrelation calculations (Chen et al., 2012). However, we have to admit that although the cost of sequencing technologies has dramatically decreased, sequencing with different platforms or multiple time points is unlikely to be performed for more than economic reasons only. In addition, the large volume of omics data sets will require substantial investments in data storage and management.

Topol (2014) rightfully indicated that individualized medicine needs translating large-scale omics data sets into useful knowledge. The approaches of omics data analysis can be roughly categorized as bioinformatics and network-based. Bioinformatics-based approaches often use statistical techniques to assess significant difference or association in the omics data. Their biological interpretation mainly relies on annotations in the community databases. Due to the chosen scope of this review, we are not going into details of these approaches. Network-based approaches, on the other hand, are mainly used to integrate multi-omics simultaneously and the network itself is subsequently used to explore biological insights. In general, network-based approaches first reconstruct biased or unbiased networks *in silico*, and then use the reconstructed network to interpret the omics data. A biased network indicates that prior biological knowledge is incorporated, whereas an unbiased network is purely data-driven.

Network-based approaches enable us to link genotype to phenotype, and vice versa. The constructed networks can be viewed as maps, in which we can locate GWAS results and improve our understanding the roles of genetic/epigenetic alterations in disease predisposition (Califano et al., 2012; Ghiassian et al., 2015). At the same time, these maps can also help us tracking back molecular mechanisms of given clinical phenotypes. Like what has been shown by Bartel et al. (2015), the “human blood metabolome-transcriptome interface,” a network constructed based on the correlation between serum metabolomes and whole blood transcriptomes of 712 subjects, can identify active pathways/modules with concentrations of blood cholesterol and triglycerides. In the next sections, we focus on three types of network-based approaches, namely gene regulatory network, protein-protein interaction networks, and genome-scale metabolic modeling and discuss them in a schematic manner: i.e., (1) definition and generation; (2) usage and results; (3) strength and weakness. We also discuss their applicability for individualized medicine.

## 3. Gene regulatory networks

### 3.1. What are gene regulatory networks?

Thousands of gene products are produced from the human genome to support cell function and survival. The protein-coding genes can induce protein synthesis, whereas the non protein-coding genes encode noncoding RNAs (ncRNAs) as their gene products. Gene regulatory networks (GRNs) ensure proper levels of gene products present at the right time in the cell (Karlebach and Shamir, 2008). In the GRN, nodes represent the genes and edges indicate the interactions between gene products.

### 3.2. How are GRNs generated?

Similar to gene coexpression networks, GRNs are statistically inferred from a large number of gene expression data sets. However, gene coexpression networks and GRNs are fundamentally different from each other. Pearson's correlation coefficient is used to infer coexpression networks, meaning that there is always a direct interaction for any pair of genes when their expressions are statistically correlated (Stuart et al., 2003). In contrast, GRNs are inferred mainly based on mutual information, which explicitly specifies direct or indirect interaction for each pair of genes. Mutual information defines how much information one random variable X provides about another random variable Y (Cover and Thomas, 2006). For GRNs, the random variables refer to the gene expression levels. Almost all major algorithms developed for GRN inference are mutual information-based and include ARACNe (Algorithm for the Reconstruction of accurate Cellular Networks) (Basso et al., 2005; Margolin et al., 2006), CLR (Context Likelihood of Relatdeness) (Faith et al., 2007), MRNET (Meyer et al., 2007), RN (Relevance Network) (Butte and Kohane, 2000), C3Net (Altay and Emmert-Streib, 2010a), and BC3Net (de Matos Simoes and Emmert-Streib, 2012). Different inference algorithms above were used to reconstruct human B cell GRNs and found the networks contained consistent biological information (Altay and Emmert-Streib, 2010b; de Matos Simoes et al., 2013). We refer readers to a recent review (Emmert-Streib et al., 2014) for more general concepts of GRN inference and applications. In this review, we focus on ARACNe since it is the most widely used method. ARACNe makes use of two steps to infer a genome-wide GRN (Basso et al., 2005). First, ARACNe assesses all the pair of genes by calculating their mutual information. Then, ARACNe discriminates whether the pair of genes are directly linked or separated by any other genes through applying a well-known property of mutual information called the data processing inequality (Basso et al., 2005; Cover and Thomas, 2006).

### 3.3. What are GRNs used for?

The rationale of the GRN lies in the idea that genetic/epigenetic alterations contribute to disease phenotypes by inducing changes in a finite number of regulatory bottlenecks, i.e., transcription factors (TFs; Lefebvre et al., 2010; Califano et al., 2012). ARACNe-inferred GRNs are used for identification of the crucial TFs (also called master regulators) that affect the transition from healthy to diseased states and vice versa. The identified master regulators then serve as starting points to search for the driver genetic/epigenetic alterations upstream.

### 3.4. What has come out?

Lefebvre et al. (2010) applied ARACNe to infer a human B-cell specific GRN from 254 B-cell microarray expression profiles representing 24 distinct phenotypes. The ARACNe-inferred B-cell GRN was subsequently used to identify *MYB* and *FOXM1* as the master regulators of B-cell proliferation. Similarly, an ARACNe-inferred glioblastoma GRN was created and used by Chen et al. (2014) to identify two master regulators, *C/EBP*β and *C/EBP*δ that are known to be involved in mesenchymal subtype of glioblastoma patients (Carro et al., 2010). Furthermore, by combining the genetic variants from the same glioblastoma patients, the authors identified that *KLHL9* deletions are upstream of the two identified master regulators and act as driver mutations (Chen et al., 2014).

### 3.5. Strengths and weaknesses

One of the major advantages of ARACNe-inferred GRNs is that with whole genome microarray or total RNA sequencing, the entire genome can actually be included in the ARACNe-inferred GRNs. Moreover, since it has been shown that the interactions inferred by the ARACNe algorithm are very likely to represent real biophysical and biochemical interactions (Basso et al., 2005; Lefebvre et al., 2010), ARACNe-inferred GRNs are suitable to explore all the possible interactions related to ncRNAs. This represents an important feature of ARACNe-inferred GRNs, as more or less 90% of the human genome is being transcribed, but only about 3% encodes protein. It is known that long noncoding RNAs (lncRNAs) can interact with DNA and proteins (Quinodoz and Guttman, 2014), and some lncRNA interactions are related to human diseases. For example, Hirata et al. (2015) reported that interaction between lncRNA MALAT1 and histone-lysine N-methyltransferase EZH2 is involved in renal cell carcinoma.

The major drawback of ARACNe is that a large number (≥100) of gene expression profile data covering a broad range of phenotypes is required to infer the target GRNs (Basso et al., 2005; Margolin et al., 2006). This is indeed necessary to explore a significant range of gene expression dynamics in order to obtain adequate mutual information for inferring GRNs (Margolin et al., 2006). Obviously, in practice it is costly and time-consuming.

## 4. Protein-protein interaction networks

### 4.1. What are protein-protein interaction networks?

Proteins exert their function through interactions with other molecules (e.g., DNA, RNA, proteins, and metabolites). For instance, signal transduction is mediated through protein-protein interactions (PPIs), whereas gene expression (transcription factor-DNA) and metabolism (enzyme-substrate interaction) are mediated by protein-DNA and protein-metabolite interactions, respectively (Sevimoglu and Arga, 2014). PPIs can also refer to formation of dimers, multi-protein complexes or supramolecular assemblies (e.g., actin filaments). Since some proteins are shared by different PPIs, individual PPIs are interconnected. In the PPI network, nodes represent genes whereas edges refer to physical interactions of the respective proteins.

### 4.2. How are PPI networks generated?

There are three main resources of generic human PPI networks. The first resource is from the literature mining. We listed six primary databases (Table 2) that store and combine experimentally supported PPIs from small-scale studies. The second resource is derived from large-scale yeast-two-hybrid (Y2H) screening. In 2005, the first generation of Y2H-based human PPI network, HI-I-05, was introduced and included 2700 high-quality binary PPIs among 1705 proteins (Rual et al., 2005; Stelzl et al., 2005). In 2014, the second generation of Y2H-based human PPI network, HI-II-14, was released (Rolland et al., 2014). This time 13,944 PPIs were identified among 4303 proteins. Both HI-I-05 and HI-II-14 can be downloaded (http://interactome.dfci.harvard.edu/H_sapiens/). In addition to the Y2H system, affinity-purification mass spectrometry (AP-MS) is also developed to profile PPIs in human cells (e.g., human HEK293T, Huttlin et al., 2015). Compared to Y2H which is mainly used to identify binary interactions between two proteins, AP-MS is more focusing on deciphering the composition of protein complexes. The third resource of the human PPI network is the computational prediction, in which machine learning algorithms are applied to calculate the likelihood of interactions between two proteins based on the known interactions in the databases (Table 2). STRING (Search Tool for the Retrieval of Interacting Genes, http://string-db.org/) (Snel et al., 2000) is such a web-server including known and predicted protein interactions of over 2000 organisms. In addition to STRING, databases, such as PIPs (http://www.compbio.dundee.ac.uk/www-pips/) (McDowall et al., 2009) and hPRINT (human Predicted Protein Interactome) (Elefsinioti et al., 2011) also predict PPIs without priori experimental evidence. The hPRINT results can be retrieved in STRING as well (Franceschini et al., 2013).

**Table 2:** **Primary sources of protein-protein interactions**.

**Name**	**Link**	**References**
HPRD	http://www.hprd.org/	Keshava Prasad et al., 2009
IntAct	http://www.ebi.ac.uk/intact/	Orchard et al., 2014
MINT	http://mint.bio.uniroma2.it/mint/Welcome.do	Licata et al., 2012
DIP	http://dip.doe-mbi.ucla.edu/dip/Main.cgi	Xenarios et al., 2002
BioGRID	http://thebiogrid.org/	Stark et al., 2006
PDB	http://www.rcsb.org/pdb/home/home.do	Berman et al., 2000

Human proteome studies have shown distinct proteome profiles in different cell and tissue types (Kim et al., 2014; Uhlén et al., 2015). This makes it necessary to specify PPI networks in the target cell and tissue (Schaefer et al., 2013). TissueNet database (http://netbio.bgu.ac.il/tissuenet/) provides such context-specific PPI networks for 16 human tissues (Barshir et al., 2013). A generalized way to construct such context-specific PPI networks is introduced by Magger et al. (2012), who developed a specific algorithm integrating context-specific gene expression data (proteomics or transcriptomics). Gene expression data are used to assess the probability of PPIs in the generic PPI network. If a gene is not expressed, the algorithm can either remove the gene from the generic PPI network or reduce the weight of the interactions associated with the gene.

### 4.3. What are PPI networks used for?

Human PPI networks are used to identify genes, proteins and subnetworks associated with diseases (Sevimoglu and Arga, 2014). They are also used to systematically characterize PPI network perturbations associated with disease mutations. The PPI network perturbations include complete loss of gene products or alteration of PPI arrangement (Zhong et al., 2009; Sahni et al., 2013).

### 4.4. What has come out?

Goehler et al. (2004) generated a PPI network for Huntington's disease by using the Y2H. From there, they identified GIT1, a G protein-coupled receptor kinase-interacting protein, which directly interacts with huntingtin and turns out to enhance huntingtin aggregation. Based on the generic human PPI network derived from HPRD (Human Protein Reference Database; Keshava Prasad et al., 2009), Jia and Zhao (2014) focused on PPI subnetworks that contain multiple genes frequently mutated in lung adenocarcinoma and melanoma patients. The results showed that the driver mutations interrupted the PPIs that are involved in signaling pathways (e.g., EGF receptor signaling pathway) and biological processes (e.g., DNA repair systems; Jia and Zhao, 2014). Based on the Y2H protein interaction assays, Sahni et al. (2015) reported that common SNPs from healthy subjects rarely affected PPIs, but around 60% of human disease-associated missense mutations perturbed PPIs. Furthermore, they also noticed that different mutations in the same gene can influence different PPIs.

### 4.5. Strengths and weaknesses

Unlike the ARACNe-inferred GRNs, in which the interactions are statistically inferred from the gene expression levels, PPI networks derived from the literature or Y2H screening are experimentally supported. Therefore, perturbations in PPI networks can be used with confidence to elucidate the molecular basis of diseases as described in the examples given above.

A weakness of the PPI networks is incomplete coverage. According to the up-to-date GENCODE release 23 (http://www.gencodegenes.org/), there are 19,797 protein-coding genes in the human genome. The number of genes covered by the most comprehensive human PPI network, HI-II-14 (Rolland et al., 2014), is only 3146 which suggests that there is still a long way to go. In addition, PPIs are often evaluated under unphysiological conditions, leading to false positive and negative PPIs included in generic PPI networks (Schaefer et al., 2013). Kuchaiev et al. (2009) reported that the false positive and negative rate of Y2H could be as high as 64 and 71%, respectively.

## 5. Genome-scale metabolic models

### 5.1. What are genome-scale metabolic models?

Metabolites are implicated in maintenance of cellular functions and production of building blocks (e.g., purines and pyrimidines) for macromolecular biosynthesis. Computational biologists have reconstructed all metabolic reactions into one large network and name it “genome-scale metabolic model.” GEMs and GSMMs are typically used as abbreviations in the literature.

### 5.2. How are GEMs generated?

In general, GEMs are constructed by using enzyme-mediated reactions, transporters and intermediary metabolites (Bordbar et al., 2014). The first landmark studies in this field emerged in 2007 when Recon1 (Duarte et al., 2007) and EHMN (Edinburgh Human Metabolic Network) (Ma et al., 2007) were manually reconstructed based on genomic and experimental data in the literature. These two human metabolic networks were merged into the HMR (Human Metabolic Reaction) database (Agren et al., 2012). In 2010, a human hepatocyte-specific metabolic network, HepatoNet1, was reconstructed based on experimental evidence for presence of metabolic reactions in human hepatocytes (Gille et al., 2010). The experimental evidence was manually curated based on information from over 1500 scientific articles. In 2013, the continuing development of Recon1, EHMN, and HepatoNet1 leads to the release of Recon2 (Thiele et al., 2013). A year later, another reconstruction of human hepatocyte-specific metabolic network, iHepatocytes2322, together with a new release of the Human Metabolic Reaction database, HMR2, were published (Mardinoglu et al., 2014).

Recon2 (Thiele et al., 2013) and HMR2 (Mardinoglu et al., 2014) represents all current knowledge of global human metabolism. Since different cell/tissue types may harbor synonymous enzymes to catalyze the same reaction and different metabolic pathways may result in the same product (Uhlén et al., 2015), it is important to reconstruct cell/tissue type specific GEMs to characterize the metabolism of target cells and tissues. For this purpose, algorithms, such as tINIT (task-driven Integrative Network Inference for Tissues) (Agren et al., 2014), GIMME (Gene Inactivity Moderated by Metabolism and Expression) (Becker and Palsson, 2008), and mCADRE (metabolic Context-specificity Assessed by Deterministic Reaction Evaluation) (Wang et al., 2012) are used to generate cell/tissue type specific GEMs from the generic GEMs (e.g., Recon2 or HMR2). These algorithms use abundances of transcripts and proteins to estimate the probability of presence of enzymes in the generic GEMs. We refer readers to an excellent review (Machado and Herrgård, 2014) for more details on the differences between the various algorithms.

### 5.3. What are GEMs used for?

Human GEMs, especially cell/tissue type specific GEMs, are mainly used as scaffolds to analyze transcriptomics data obtained from patient samples, in order to identify the metabolic pathways and metabolite biomarkers that are related to disease pathogenesis.

### 5.4. What has come out?

Using the tINIT algorithm with proteomics and transcriptomics data of human myocytes, Väremo et al. (2015) reconstructed a myocyte-specific GEM, iMyocytes2419, which made it possible to reveal that type 2 diabetes patients show extensive transcriptional changes in reactions involved in pyruvate oxidation, branched-chain amino acid catabolism, and tetrahydroflate metabolism. Mardinoglu et al. (2014) applied iHepatocytes2322 and their previously developed Reporter Metabolite algorithm (Patil and Nielsen, 2005) to analyze transcriptomics data of patients with non-alcoholic fatty liver disease, and identified that concentrations of chondroitin and heparan sulfates may represent novel biomarkers for diagnosing non-alcoholic steatohepatitis. Similar GEM-based analyses have been performed to study diseases such as, Alzheimer's disease (Lewis et al., 2010), obesity (Mardinoglu et al., 2013), and cancer (Agren et al., 2014; Yizhak et al., 2014).

### 5.5. Strengths and weaknesses

In our opinion, the major advantage of GEMs is that it allows to study global metabolic flux distributions. The rate of the metabolic reactions in a pathway (metabolic flux) is determined by many aspects, such as protein concentration, protein interaction (signal transduction), enzyme kinetics and metabolite concentrations (Winter and Krömer, 2013). Therefore, metabolic fluxes can be considered as the ultimate outcome of cellular regulation at different levels (Nielsen, 2003). When listing all the reactions as well as their corresponding flux values under a particular condition, one can construct a metabolic flux distribution that represents a particular cellular phenotype in detail.

Currently, ^13^C stable isotope labeling is the most popular experimental method to measure *in vivo* fluxes (Blank and Ebert, 2013). By performing ^13^C fluxomic experiments, Murphy et al. (2013) noticed that different levels of oncoprotein MYC can induce distinct metabolic flux distributions in P493-6 B cells. They showed that high MYC cells as rely more heavily on amino acids and mitochondrial oxidative metabolism than low MYC cells. ^13^C fluxomics also revealed distinct metabolic flux distributions in different cell lines. Niklas et al. (2011) reported that human neuronal AGE1.HN cells had lower flux rates (around 2.3% of the glucose uptake flux) in the pentose phosphate pathway than other cell lines, such as HEK-293 cells (15%) and hybridoma cells (20%). These ^13^C fluxomic studies illustrate that various biological conditions can induce distinct metabolic flux distributions.

However, ^13^C fluxomics cannot deliver us a complete picture of flux distributions in the metabolic network, since only a small number of reactions can be measured. Here, GEMs provide a means to estimate metabolic flux distributions under different conditions relying on a limited number of exchange fluxes, i.e., fluxes of substrates entering the cells and the fluxes of metabolites that are secreted from the cells. It is beyond the scope of this review to explain the related mathematical theory, but we recommend the article by Rossell et al. (2011), in which they formulated how to compute complete set of fluxes from the exchange fluxes.

Bordel et al. (2010) introduced a random sampling method which can calculate means and standard deviations for each flux in the GEM under different experimental conditions, when a limited number of measurements of exchange fluxes are given. By integrating changes in gene expression between different conditions, metabolic reactions can be classified as either transcriptionally regulated (significant changes in both flux and gene expression levels), post-transcriptionally regulated (significant changes in gene expression levels but not flux), or metabolically regulated (significant changes in flux but not gene expression levels). This random sampling method was applied together with the adipocyte-specific GEM, iAdipocytes1809, and helped identifying the fluxes of glucose uptake, fatty acids uptake, oxidative phosphorylation, mitochondrial and peroxisomal β-oxidation, fatty acid metabolism and tricarboxylic acid cycle as being differentially down regulated in obese subjects (Mardinoglu et al., 2013). Gavai et al. (2015) developed a novel algorithm called Lsei-FBA (Lesat-squares with equalities and inequalities Flux Balance Analysis), and identified the fluxes of glycolysis and oxygen uptake as being decreased in brains of Alzheimer's disease patients (29 and 46%, respectively) compared to healthy subjects. Similar to the random sampling method, Lsei-FBA also requires tissue-specific GEMs, and measurements of gene expression as well as exchange fluxes.

The second biggest advantage of GEMs is that up to now it is currently the only platform that can integrate genomics, transcriptomics, proteomics, metabolomics, and fluxomics data. Yizhak et al. (2010) integrated quantitative proteomics and metabolomics with a GEM of the human erythrocyte, and predicted metabolic flux distributions in red blood cells. The flux distribution predictions were found to be consistent with the simulations made by a detailed kinetic model of human red blood cells. Bordbar et al. (2012) analyzed transcriptomics, proteomics, and metabolomics data sets of LPS-stimulated RAW 264.7 cells with a GEM of the RAW 264.7 cell line, and identified a suppressive role for *de novo* nucleotide synthesis in macrophage activation.

Last but not the least, it has been shown by Uhlén et al. (2015) that the minimum requirement of generating a cell/tissue type specific GEM is a single RNA sequencing profile.

Naturally, GEMs also have their limitations. First of all, although novel metabolite biomarkers for various diseases have been predicted by using cell/tissue type specific GEMs, few of them have been validated in humans, because of either technical limitation of measuring the metabolites in question or difficulty of accessing the patient materials. Secondly, since GEMs focus on metabolic enzyme-coding genes, reactions and pathways, GEMs cannot be used to study signal transduction pathways. Lastly, GEMs do not contain detailed kinetics of enzymes and produce metabolic flux distributions only under steady state conditions.

## 6. The future of individualized medicine

### 6.1. Role for GRNs

Regarding individualized medicine, longitudinal transcriptomics derived from cells/tissues of an individual including healthy and diseased states are the ideal resources to assemble an individualized GRN. Zoppoli et al. (2010) introduced TimeDelay-ARACNe to infer GRNs specifically from time-course data. Such ARACNe-inferred GRN provides a personalized map, with which one can locate the genetic mutations identified in the one-dimensional genome sequences in a multi-dimensional network. By integrating gene differential expression information between healthy and diseased states, one can also identify the crucial transcription factors controlling the phenotype transition. Taken together with the network location information, one can make the most of the personal genomic information and further prioritize the damaging effect of genetic mutations.

### 6.2. Role for PPI networks

PPI networks are proposed playing a role in buffering the impact of genetic mutations and environmental challenges (Forler et al., 2014; Garcia-Alonso et al., 2014). This opinion has been investigated by Garcia-Alonso et al. (2014), who built up a human PPI network by merging generic PPI networks derived from three public databases (BioGRID, Stark et al., 2006, IntAct, Orchard et al., 2014, and MINT, Licata et al., 2012). They used the reconstructed PPI network to study the effect of genetic variants predicted to be deleterious in the subjects participating in the 1000 Genomes Project, 252 healthy Spanish individuals, and 41 chronic lymphocytic leukemia patients. Interestingly, most of the potentially damaging genetic variants in healthy individuals were located in peripheral regions of the PPI network and did not really perturb the structure of the PPI network. However, when investigating the somatic variants that were predicted to be deleterious in chronic lymphocytic leukemia patients, they noticed that these mutations tended to be in internal regions of the PPI network. The above study indicates that PPI networks can help to identify whether genetic variants may be disrupting PPIs and hence may be important in explaining diseases.

### 6.3. Role for GEMs

GEMs have already been used successfully for individualized medicine. Agren et al. (2014) reconstructed personalized GEMs for 6 hepatocellular carcinoma patients based on proteomics data, and used these models to identify potential anticancer drug targets for the individual patients. Yizhak et al. (2014) reconstructed personalized GEMs for breast and lung cancer patients based on gene expression measurements obtained from biopsy samples. These personalized GEMs were used to predict the cancer cell growth rate, which was used to infer patient survival.

For successful individualized medicine, it should be realized that it is important to integrate information of cell/tissue type specific GEMs, in an attempt to capture whole-body metabolism. Urine, plasma, and serum are the most common samples from human subjects for diagnostic purpose (Nicholson et al., 2012). Metabolic measurements based on these samples are the results of the crosstalk of many organs and can be regarded as serving the readouts of whole-body metabolism.

Bordbar et al. (2011) build a multi-tissue GEM by integrating adipocyte, hepatocyte and myocyte-specific GEMs via a blood compartment. The assembled multi-tissue GEM was used to study the metabolic differences between non-type 2 diabetes obese and type 2 diabetes obese individuals. They reported that type 2 diabetes obese individuals lack activity in reactions catalyzed by lactate dehydrogenase, catalase and cysteine dioxygenase, comparing to the non-type 2 diabetes obese subjects. Besides integrating metabolism of different tissues and cells, the human gut microbiome is also considered important for whole-body metabolism (Mardinoglu and Nielsen, 2015). Shoaie et al. (2015) reconstructed five GEMs for five representative bacteria in the human gut, including *Bacteroides thetaiotanmicron, Eubacterium rectale, Bifidobacterium adolescentis, Faecalibacterium prausnitzii*, and *Ruminococcus bromii*. These GEMs were used to study 45 overweight and obese individuals who were subjected to an energy-restricted, high-protein diet intervention for 6 weeks. The authors reported that the diet intervention decreased the gut microbiota production of short chain fatty acids (acetate, butyrate, and propionate) and amino acids (e.g., alanine, proline and glycine etc.).

### 6.4. Concluding remarks

Due to the central role of the interactome in cellular functions, we think that the roadmap of individualized medicine is moving from human genomes to interactomes. However, construction of a complete human interactome is extremely complex and it might take at least another decade (Menche et al., 2015). This review shows that GRNs, PPI networks, GEMs can characterize part of the interactome in cells. Integrating different type of networks may contribute to better understanding of the interactome, and ultimately realizing true individualized medicine.

## Author contributions

XZ wrote the manuscript. JK edited the manuscript. AG edited the manuscript.

## Funding

This work was supported by grants CVON-Genius (CVON2011-19) and RESOLVE (FP7 305707).

### Conflict of interest statement

The authors declare that the research was conducted in the absence of any commercial or financial relationships that could be construed as a potential conflict of interest.
